# Silencing of miR-483-5p alleviates postmenopausal osteoporosis by targeting SATB2 and PI3K/AKT pathway

**DOI:** 10.18632/aging.202552

**Published:** 2021-02-17

**Authors:** Fujiang Zhao, Yier Xu, Yulong Ouyang, Zhexu Wen, Guihao Zheng, Ting Wan, Guicai Sun

**Affiliations:** 1 Department of Orthopaedics, Taizhou Central Hospital (Taizhou University Hospital), Taizhou 318000, China; 2 Laboratory of Pharmacology, Research and Development Center of Harbin Pharmaceutical Group, Harbin 150025, China; 3 Medical College of Nanchang University, Nanchang 330006, China; 4 Department of Orthopaedics, The First Affiliated Hospital of Nanchang University, Nanchang 330006, China

**Keywords:** postmenopausal osteoporosis, miR-483-5p, SATB2, PI3K/AKT pathway

## Abstract

Postmenopausal osteoporosis (PMOP) poses a significant threat to women’s health worldwide. However, detailed molecular mechanism and therapeutic strategy for PMOP remain insufficient. Accumulating evidence suggests that miR-48-5p is implicated in the pathogenesis of osteoporosis. The present study aimed to determine the role and mechanism of miR-483-5p in PMOP. Results from PMOP patients demonstrated that miR-483-5p was up-regulated and SATB2 was down-regulated. Luciferase reporter assay identified SATB2 as a direct target gene of miR-483-5p. Experiments in MC3T3-E1 cells indicated that miR-483-5p mimic markedly inhibited cell viability as well as the expressions of OPG, RUNX2 and BMP2. And miR-483-5p inhibitor, SATB2-overexpressed lentiviruses (Lv-SATB2) or LY294002 (PI3K/AKT inhibitor) significantly reversed the above results. Similarly, PI3K/AKT signaling was activated by miR-483-5p mimic, and was inhibited in miR-483-5p inhibitor, Lv-SATB2 or LY294002 treated cells. *In vivo* experiments showed that miR-483-5p inhibitor significantly increased the bone mineral density and biomechanical parameters of femurs in ovariectomized (OVX) rats by targeting SATB2. In addition, the osteogenic differentiation and PI3K/AKT signaling were also regulated by miR-483-5p-SATB2 axis. Taken together, our findings indicated that miR-483-5p contributed to the pathogenesis of PMOP by inhibiting SATB2 and activating PI3K/AKT pathway. MiR-483-5p/SATB2 could be selected as a potential therapeutic target for PMOP.

## INTRODUCTION

Osteoporosis is featured by a depletion in bone mass and micro-architecture degeneration, which generally raises the risk of bone fragility and fractures [1]. Osteoporosis has been reported to result in one and a half million fractures per year in the United States, with the most affected individuals being postmenopausal women [2]. Postmenopausal osteoporosis (PMOP) is considered a primary type of osteoporosis in clinical practice. Postmenopausal women are more likely to suffer from osteoporosis due to multiple factors, including aging, estrogen and calcium levels [3]. A white woman in her 50s has a 50% lifetime risk of osteoporotic fracture, which is a significant burden on public healthcare systems [4]. Although there are several medications for the PMOP treatment, a gold standard treatment still needs to be established [5]. Thus, there is an immediate need to understand the detailed molecular mechanism and identify potential biomarkers underlying PMOP so that a novel therapeutic strategy can be developed.

MicroRNA (miRNA) is a kind of small non-coding RNA formed by 21~25 nucleotides that exerts important functions in several biological pathways, including cell apoptosis, differentiation and proliferation, by regulating the protein expression through post-transcriptional gene silencing [6]. More than 30 % of the human genes are predicted to be regulated by miRNAs. So far, over 2,500 miRNAs have been identified in human beings [7]. Numerous differentially expressed miRNAs have been identified as related to molecular pathogenesis of PMOP in recent years [7], including miR-133a, miR-140-5p, miR-542-3p, miR-194-5p, and others [8, 9]. The intron 2 of insulin-like growth factor 2 contains the miR-483, which generates two mature isoforms: miR-483-5p and miR-483-3p [10]. The miR-483-5p biological function has been explored in many physiological and pathological events, including angiogenesis, cell apoptosis, tumor progression and chondrogenic differentiation [11–13]. However, the miR-483-5p role in PMOP has not been understood.

SATB2 (Special AT-rich sequence-binding protein) is a protein composed of 733 residues that has a molecular mass of 82.5 kDa [14]. It functions as an important transcription factor that is associated with nuclear matrix-attachment regions, where it activates the transcription of several genes simultaneously and has crucial roles in various developmental processes [15]. Aberrant regulation of SATB2 is closely related to several types of diseases, including cancer, Parkinson's disease, and osteoporosis [16–18]. Evidences have shown that SATB2 is regulated by a series of miRNAs. However, the miR-483-5p regulatory effect on SATB2 is unknown in PMOP.

This study aimed to uncover the miR-483-5p expression level in PMOP samples and assess the miR-483-5p effects on bone formation and resorption. The miR-483-5p target gene was identified by luciferase reporter assays. In addition, MC3T3-E1 cells and ovariectomized (OVX) rats were used to investigate the pathways underlying the miR-483-5p role in PMOP progression.

## RESULTS

### Expression of SATB2 and miR-483-5p in clinical specimens

**Figure 1:**
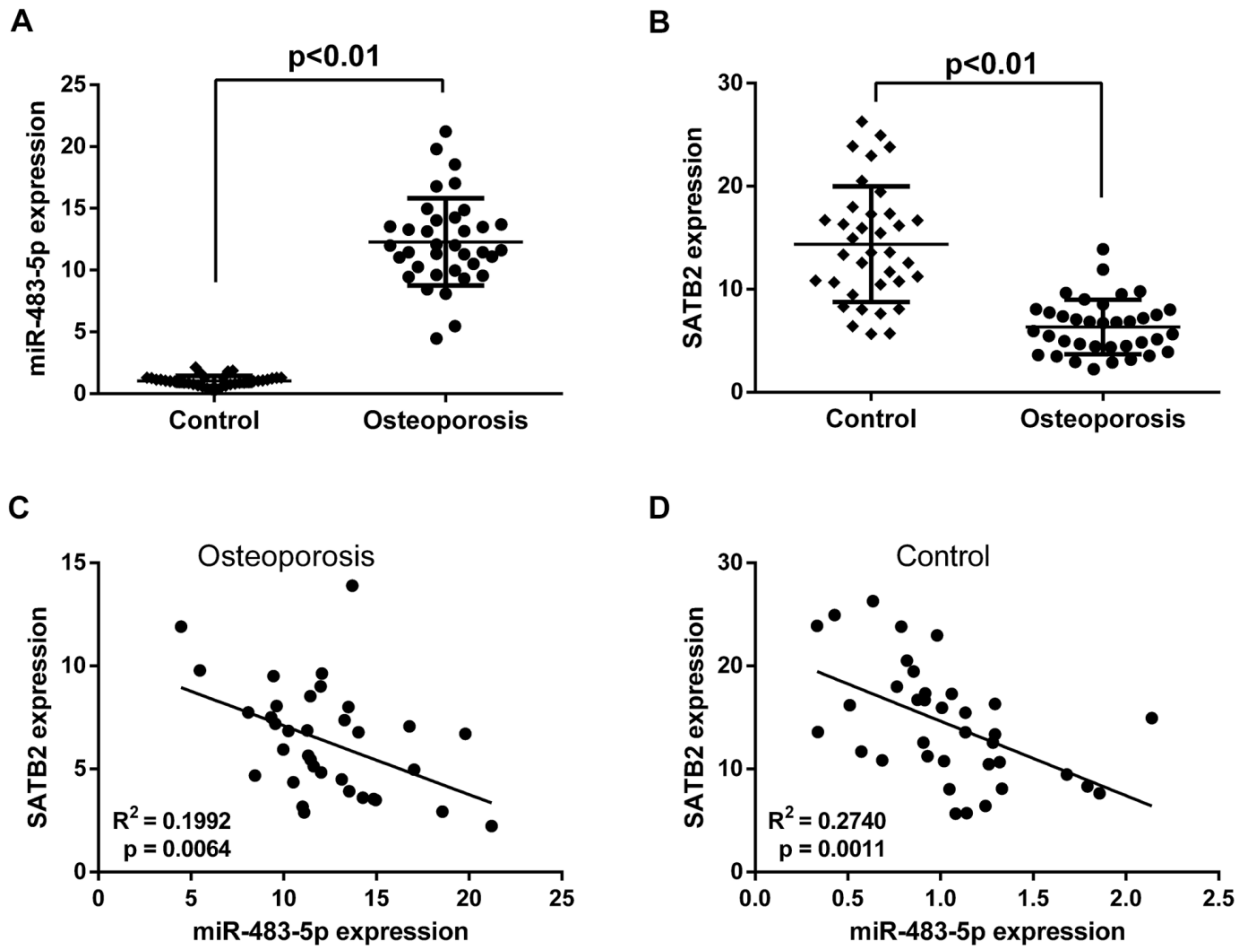
**Expressions of miR-483-5p and SATB2 in clinical samples.** (**A**) Expressions of miR-483-5p in postmenopausal patients with or without osteoporosis. (**B**) Expressions of SATB2 in postmenopausal patients with or without osteoporosis. (**C**) Pearson correlation analysis of miR-483-5p expression and SATB2 in osteoporosis samples. (**D**) Pearson correlation analysis of miR-483-5p expression and SATB2 in control samples. Data were presented as mean ± SD.

The effects of miR-483-5p and SATB2 on osteoporosis were investigated by comparing their expression levels in postmenopausal patients with and without osteoporosis. qRT-PCR results revealed that the miR-483-5p expression level was greater (Figure 1A) and that of SATB2 was lower (Figure 1B) in patients with osteoporosis compared to the control. Besides, Pearson correlation test unveiled a significant negative association between miR-483-5p and SATB2 in both osteoporosis (Figure 1C) and control patients (Figure 1D), suggesting the participation of miR-483-5p and SATB2 for the osteoporosis occurrence.

### SATB2 is a miR-483-5p direct target

**Figure 2:**
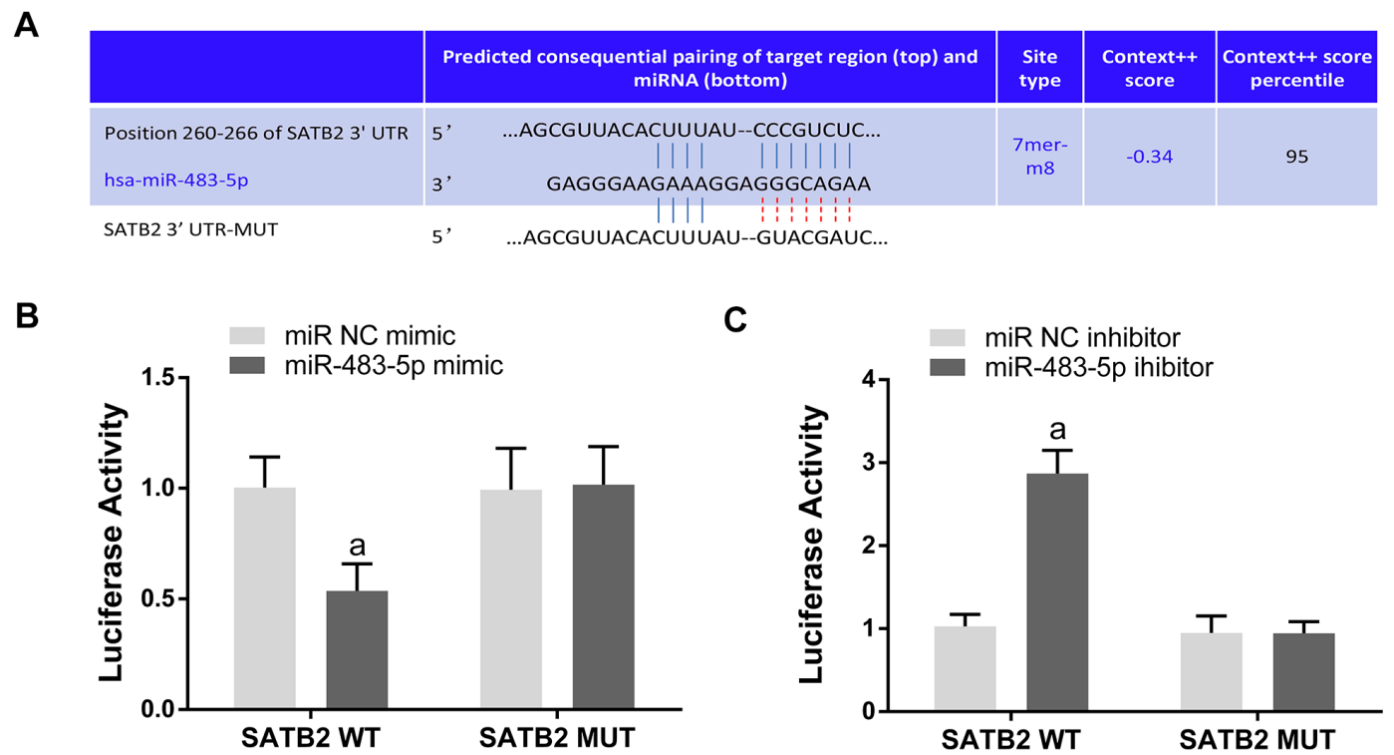
**SATB2 is a direct target gene of miR-483-5p.** (**A**) The predicted sites of miR-483-5p binding to the 3’-UTR of SATB2. (**B**) Luciferase activity was measured in miR-483-5p mimic transfected MC3T3-E1 cells. (**C**) Luciferase activity was measured in miR-483-5p inhibitor transfected MC3T3-E1 cells. Data were presented as mean ± SD. ^a^ p < 0.05 versus negative control (NC).

MiR-483-5p target genes were searched using TargetScan. SATB2 was chosen as a candidate because it has a potential miR-483-5p binding site on the 3’-UTR of its mRNA (Figure 2A). In addition, dual luciferase reporter assays were executed to validate the miR-483-5p binding sites on the SATB2 mRNA 3’-UTR. The relative luciferase activity (RLA) was significantly diminished in the STAB2 WT reporter plasmid in miR-483-5p mimic transfected MC3T3-E1 cells. In contrast, no significant differences were observed in the RLA between the miR-483-5p mimic and miR NC mimic groups with the STAB2 MUT reporter plasmid (Figure 2B). Finally, miR-483-5p inhibitor enhanced the RLA of the SATB2 reporter plasmid that contained the WT but not the MUT 3’-UTR of SATB2 (Figure 2C). These findings suggest that SATB2 is a miR-483-5p direct target in MC3T3-E1 cells.

### miR-483-5p-SATB2 axis regulates the osteogenic differentiation in MC3T3-E1 cells

**Figure 3:**
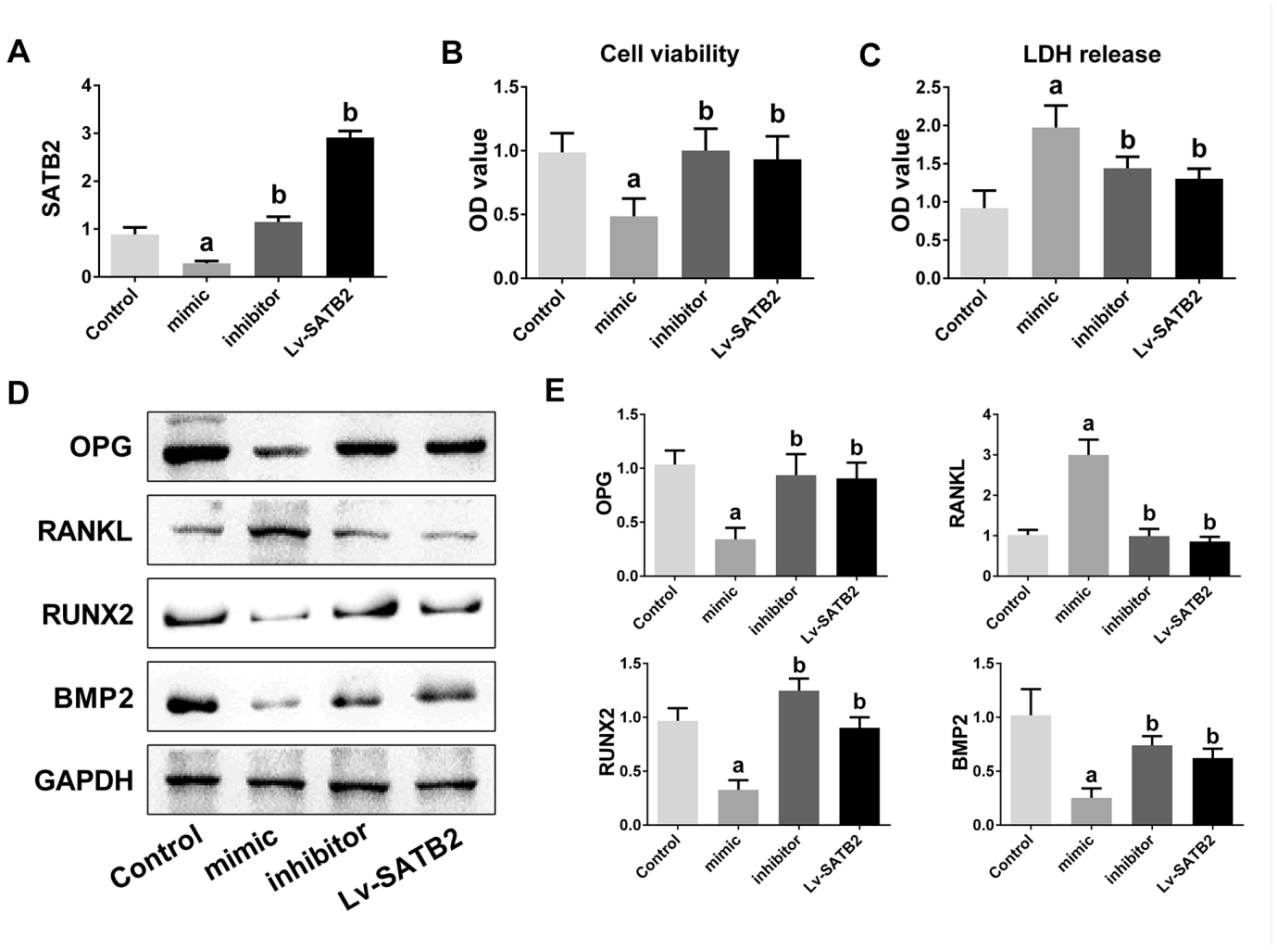
**miR-483-5p-SATB2 axis regulates the osteogenic differentiation in MC3T3-E1 cells.** (**A**) The transfection efficiency of SATB2 was detected by qRT-PCR. (**B**) Cell viability was detected 48 h after transfection. (**C**) LDH release were detected 48 h after transfection. (**D**) Western blotting results of the indicators of osteogenic differentiation in MC3T3-E1 cells. (**E**) Quantitative analysis of the optical density in (**D**). Data were presented as mean ± SD. ^a^ p < 0.05 versus control group, ^b^ p < 0.05 versus mimic group.

To investigate the biological function of miR-483-5p and SATB2 on osteogenic differentiation, MC3T3-E1 cells were co-transfected with miR-483-5p inhibitor, miR-483-5p mimic or SATB2-overexpressed lentiviruses (Lv-SATB2). qRT-PCR was employed to certify the efficiency of the transfection (Figure 3A). Cell viability was significantly decreased in miR-483-5p mimic transfected cells and recovered after transfection of miR-483-5p inhibitor or Lv-SATB2 (Figure 3B). In addition, miR-483-5p mimic enhanced the LDH level while the miR-483-5p inhibitor and Lv-SATB2 decreased the LDH release (Figure 3C). The OPG/RANKL ratio is a fundamental indicator of the bone resorption and formation balance. Western blot results unveiled that OPG expression was significantly reduced in miR-483-5p mimic transfected cells, while the RANKL expression increased in the same condition. The OPG/RANKL rate was reversed after miR-483-5p inhibitor or Lv-SATB2 treatment. Moreover, miR-483-5p mimic decreased the expression level of RUNX2 and BMP2, which were two crucial mediators of osteogenic differentiation. Likewise, miR-483-5p inhibitor and Lv-SATB2 also reversed the expression of RUNX2 and BMP2 (Figure 3D, 3E).

### PI3K/AKT signaling pathway is engaged in the osteoporosis mediated by miR-483-5p-SATB2 axis

**Figure 4:**
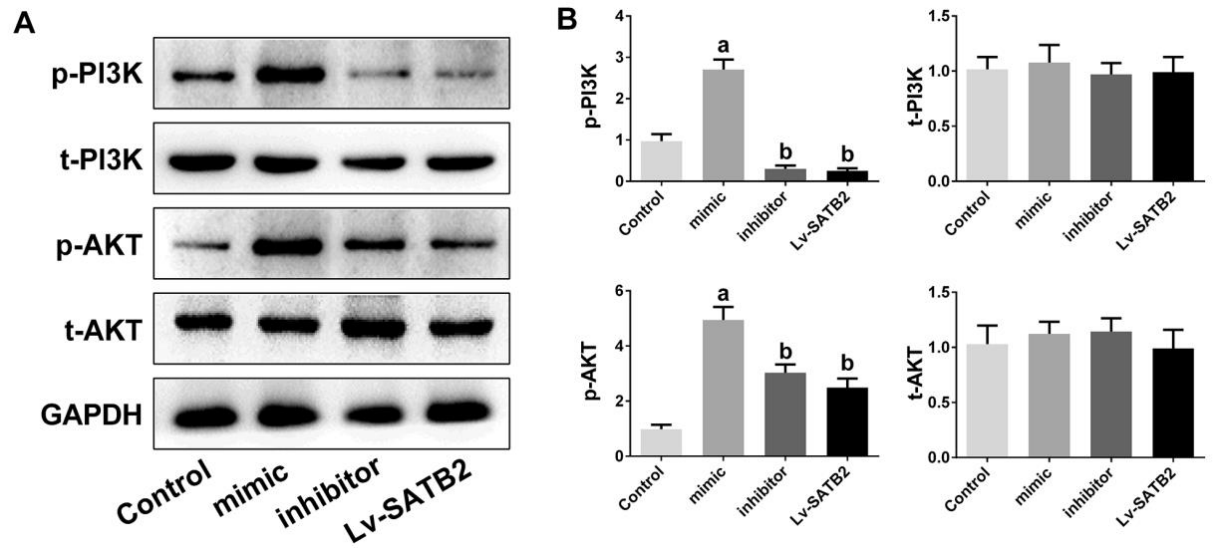
**PI3K/AKT pathway is involved in the osteoporosis mediated by miR-483-5p-SATB2 axis.** PI3K/AKT pathway is involved in the osteoporosis mediated by miR-483-5p-SATB2 axis. (**A**) Western blotting results of PI3K and AKT in MC3T3-E1 cells. (**B**) Quantitative analysis of the optical density in (**A**). Data were presented as mean ± SD. a p < 0.05 versus control group, b p < 0.05 versus mimic group.

**Figure 5:**
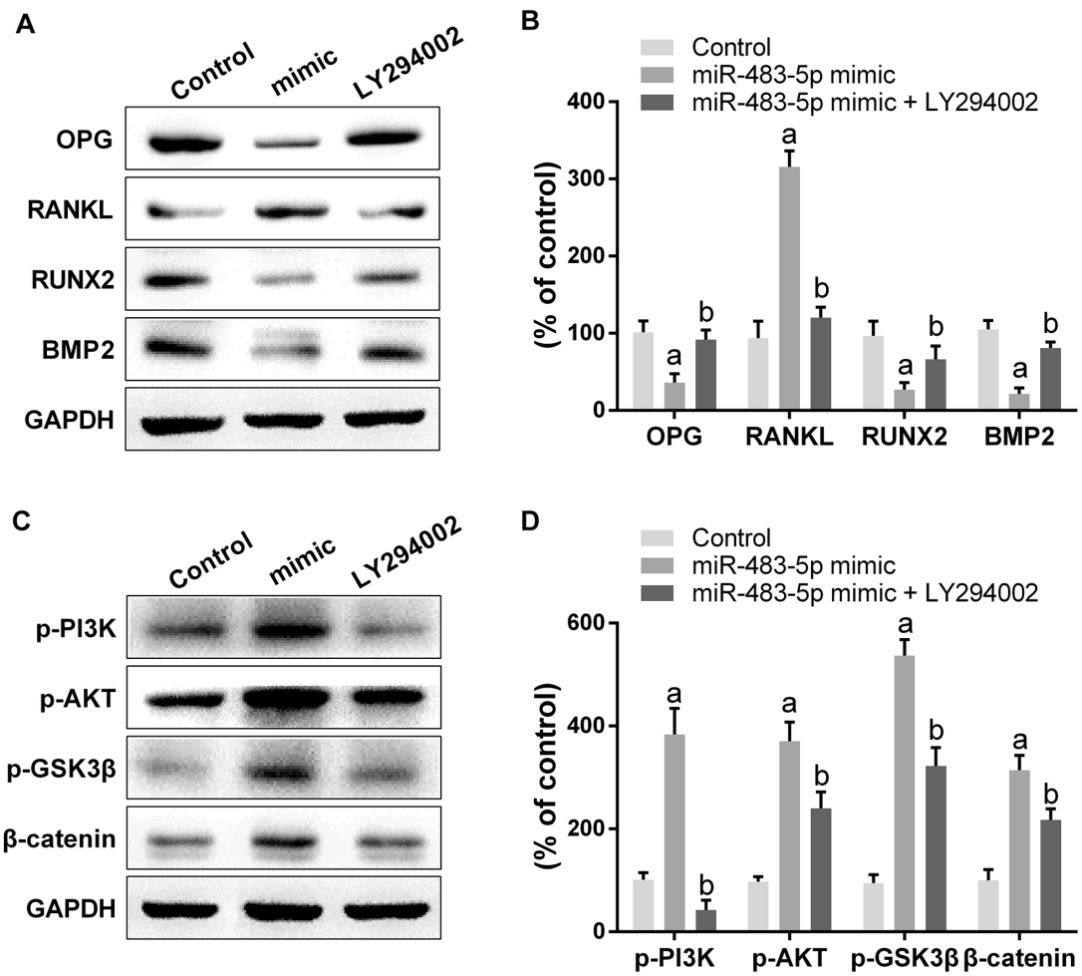
**Inhibition of PI3K/AKT pathway reversed the effect of miR-483-5p on osteoporosis.** (**A**) Western blotting results of the indicators of osteogenic differentiation in MC3T3-E1 cells. (**B**) Quantitative analysis of the optical density in (**A**). (**C**) Western blotting results of PI3K/AKT and GSK3β/β-catenin pathway in MC3T3-E1 cells. (**D**) Quantitative analysis of the optical density in (**C**). Data were presented as mean ± SD. ^a^ p < 0.05 versus control group, ^b^ p < 0.05 versus mimic group.

To examine the molecular mechanisms of miR-483-5p and SATB2 on osteoporosis, the downstream cascade PI3K/AKT pathway was analyzed. The miR-483-5p mimic markedly enhanced the PI3K (p-PI3K) and AKT (p-AKT) phosphorylation levels in comparison with the control group. However, miR-483-5p inhibition and SATB2 overexpression reversed the up-regulation of both p-AKT and p-PI3K, which had been induced by miR-483-5p mimic (Figure 4A, 4B). In addition, LY294002 was applied to inhibit PI3K/AKT signaling in the present study. LY294002 significantly reversed the expression of OPG, RANKL, RUNX2 and BMP2 (Figure 5A, 5B). The activated PI3K/AKT and GSK3β/β-catenin (downstream pathway of PI3K/AKT) signaling pathways were also inhibited by LY294002 (Figure 5C, 5D). These findings indicate that PI3K/AKT pathway is implicated in the osteoporosis development and is regulated by SATB2 in MC3T3-E1 cells.

### miR-483-5p-SATB2 axis modulates the osteoporosis progression in ovariectomized rats

**Figure 6:**
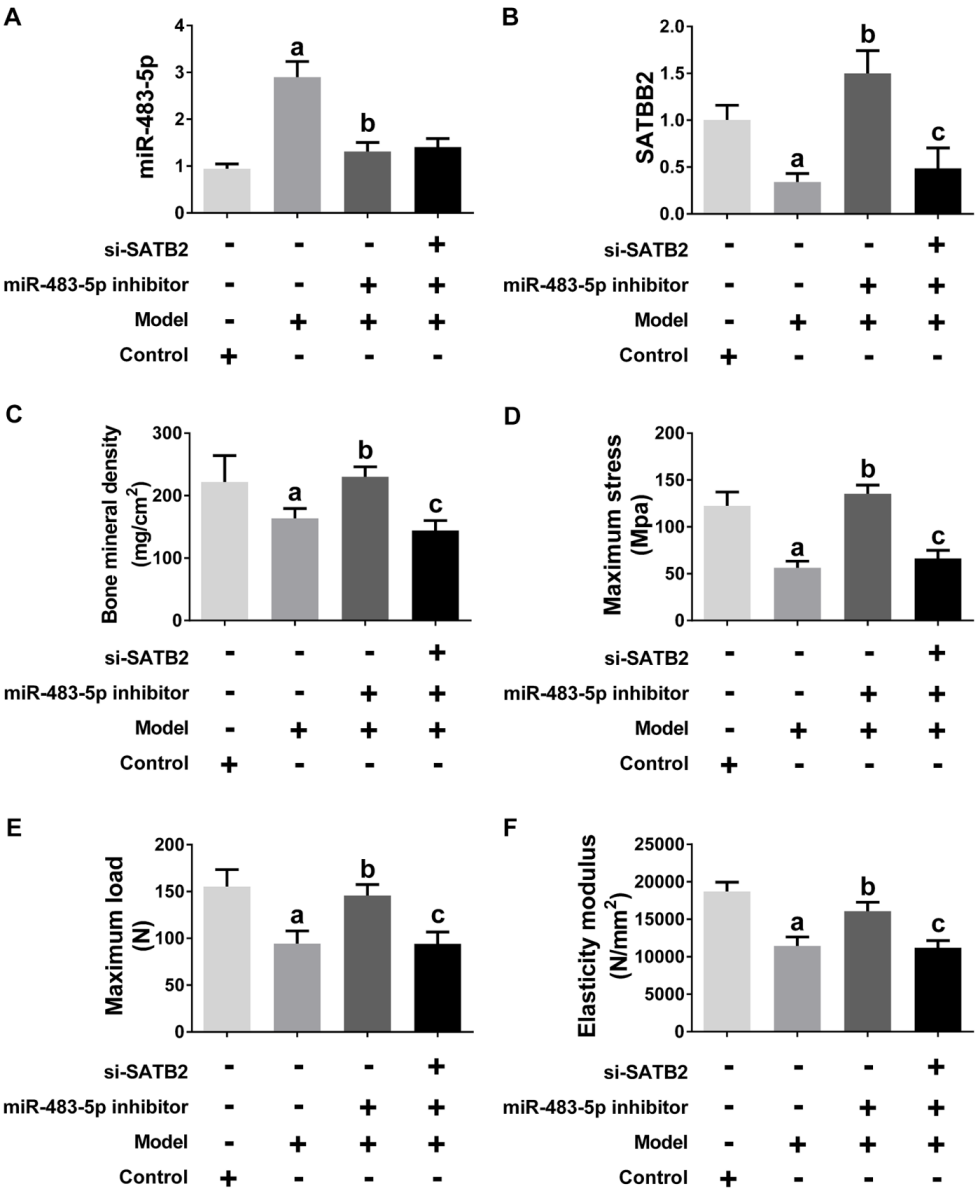
**miR-483-5p-SATB2 axis regulated the progress of osteoporosis in OVX rats.** (**A**) qRT-PCR results for miR-483-5p expression. (**B**) qRT-PCR results for SATB2 expression. (**C**) The bone mineral density (BMD) of femurs. (**D**) The maximum bending stress of femurs. (**E**) The maximum load of femurs. (**F**) The elasticity modulus of femurs. Data were presented as mean ± SD. ^a^ p < 0.05 versus control group, ^b^ p < 0.05 versus model group, ^c^ p < 0.05 versus inhibitor group.

Ovariectomized (OVX) rats were made to simulate the pathological condition of osteoporosis *in vivo*. Similar to the findings obtained in the cell experiments, the SATB2 expression was down-, while miR-483-5p expression was up-regulated in the model group, and the miR-483-5p inhibitor reversed the expression of both, subsequently (Figure 6A, 6B). siRNA-SATB2 successfully interferes with the SATB2 mRNA expression in rats (Figure 6B). The osteoporosis progress in OVX rats was also measured. In comparison with the control (sham) group, bone mineral density (BMD), elasticity modulus, maximum load and maximum bending stress were significantly decreased in OVX rats, suggesting the model was successfully established. These reduced indicators increased again after miR-483-5p inhibitor transfection (Figure 6C–6F). In addition, BMD and the values of the three biomechanical parameters decreased again after the siRNA-SATB2 injection, indicating that SATB2 is a miR-483-5p downstream gene in OVX rats.

### PI3K/AKT signaling pathway is the downstream target for the role of miR-483-5p-SATB2 axis in ovariectomized rats

**Figure 7:**
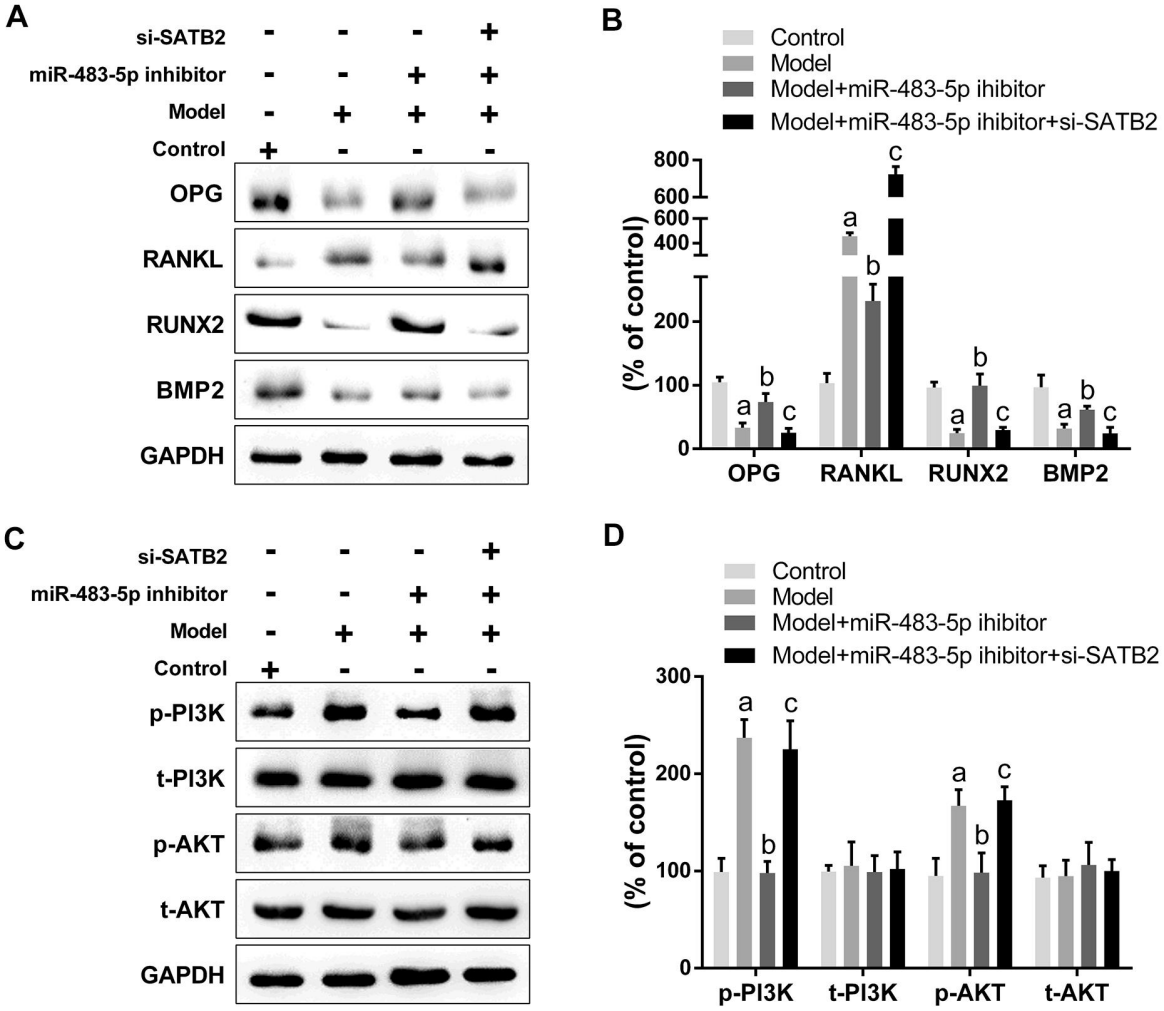
**PI3K/AKT signaling is the downstream target for the role of miR-483-5p-SATB2 axis in ovariectomized rats.** (**A**) Western blotting results of the expressions of osteogenic differentiation indicators. (**B**) Quantitative analysis of the optical density in (**A**). (**C**) Western blotting results of the expressions of PI3K/AKT signaling. (**D**) Quantitative analysis of the optical density in (**C**). Data were presented as mean ± SD. ^a^ p < 0.05 versus control group, ^b^ p < 0.05 versus model group, ^c^ p < 0.05 versus inhibitor group.

Western blot results unveiled that the expression of OPG, RUNX2, and BMP2 decreased significantly, while the RANKL expression increased in OVX rats. The miR-483-5p inhibitor reversed the expressions of these proteins compared to the model group. Remarkably, si-SATB2 reversed the changes in protein expression induced through miR-483-5p inhibitor (Figure 7A, 7B). Likewise, the AKT and PI3K phosphorylation levels were enhanced in the model group and reduced after the miR-483-5p inhibitor transfection. The siRNA-SATB2 injection successfully raised the p-PI3K and p-AKT levels (Figure 7C, 7D). These findings indicate that PI3K/AKT signaling pathway is the downstream target for the miR-483-5p-SATB2 axis role in OVX rats.

## DISCUSSION

In this study, miR-483-5p has been verified to be up- and SATB2 down-regulated in PMOP samples and a significant negative association was found between miR-483-5p and SATB2 mRNA levels in PMOP patients. In addition, SATB2 was revealed as a miR-483-5p direct target by dual luciferase reporter assays. As expected, SATB2 overexpression apparently inhibited the osteoporosis progression. Furthermore, PI3K/AKT signaling pathway was found activated by miR-483-5p/SATB2 in MC3T3-E1 cells and OVX rats.

Increasing evidence has suggested that the miRNA dysregulation is associated to various diseases, including osteoporosis [19]. More and more miRNAs are being identified as potential therapeutic targets or diagnostic biomarkers of osteoporosis [20]. MiRNA profile comparative analysis between non-osteoporotic and osteoporotic bones by miRNA arrays revealed that miR-483-5p expression in bones with osteoporosis was significantly greater than in those without osteoporosis, indicating that increasing miR-483-5p expression collaborates to the osteoporosis pathology [21]. In addition, a recent study investigated the miR-483-5p role in osteoclast formation Elevated miR-483-5p expression levels have often been found in bone tissues and serum obtained from osteoporotic patients. These findings denote that miR-483-5p participates in the osteoporosis pathogenesis through the stimulation of osteoclast differentiation [22]. Consistent with these previous studies, the miR-483-5p expression level has been also identified to be greater in PMOP patients in comparison with the control patients in the present study.

Recent studies have revealed that miRNAs are important SATB2 upstream regulators. Satb2^-/-^ mice has been reported to exhibit defects in osteoblast differentiation and function [23]. Satb2 loss leads to decreased bone mineralization, resulting in short and brittle limb bones [24]. Other studies also suggested that Satb2 regulates skeletogenesis by influencing the osteoblast differentiation, patterning and matrix formation through the modulation of osteoblast-specific genes, such as Runx2 and Atf4 [24]. Different studies have suggested that several miRNAs, including miR-187-3p, miR-4270-5p, miR-103, miR-140-5p, miR-29a-5p, are able to regulate SATB2 in various pathological conditions. Similar to miR-483-5p, SATB2 has also been shown to be found in the blood and located mainly in leukocytes and lymphocytes [25]. Here, we found that miR-483-5p is the regulator of SATB2 in PMOP patients. The miR-483-5p mimic significantly inhibited the SATB2 expression, while SATB2 overexpression successfully reversed the progress of osteoporosis induced by miR-483-5p. Moreover, SATB2 silencing has aggravated osteoporosis. These results unveil that miR-483-5p-SATB2 axis is the crucial mechanism of osteogenic differentiation.

The phosphoinositide 3-kinase (PI3K)/protein kinase B (AKT) signaling pathway is present in mammalian cells and is implicated in the modulation of cell proliferation, death and survival [26]. PI3K is a lipid kinase involved in the phosphatidylinositol-3,4,5-phosphate (PIP3) production, which in turn engages and activates the downstream molecule Akt [27]. Subsequently, the activated Akt phosphorylates several targets. Previous studies have demonstrated that AKT and its related downstream signals are essential regulatory factors for regulating endochondral ossification. As example, AKT knockout mice had been reported to show shorter bones and delayed bone ossification [28]. Moreover, Akt in conjuction with bone morphogenetic protein 2 (BMP2) modulates osteoblast differentiation from mesenchymal stromal cells [29]. AKT can also affect osteoblast survival and bone formation by retaining the Forkhead box protein O (FOXOs) in the cytoplasm [30]. Recent studies have shown that the PI3K/AKT pathway activation up-regulates the osteogenic differentiation markers gene expression, such as BMP2 and ALP, thus stimulating osteoblast differentiation and proliferation [31]. In contrast, the inhibition of PI3K/AKT signaling activity weakens the bone resportion capacity of osteoclasts [32]. In this study, AKT and PI3K phosphorylation levels were found to be significantly enhanced in MC3T3-E1 cells and OVX rats, indicating that the PI3K/AKT signaling pathway is closely associated to the osteoporosis progression. Besides, the PI3K/AKT signaling activation can be inhibited by miR-483-5p inhibitor or Lv-SATB2, and this PI3K/AKT signaling inhibition can be reversed by siRNA-SATB2. Thus, these findings show that PI3K/AKT signaling pathway is the downstream target for the miR-483-5p-SATB2 axis role.

In summary, our current study illustrated that miR-483-5p is up-regulated in postmenopausal osteoporosis and affects the osteoporosis progression by targeting SATB2 and activating the PI3K/AKT signaling pathway. These results offer new insights into the molecular mechanism underlying PMOP progression and provide theoretical support for selecting miR-483-5p/SATB2 as a target for PMOP treatment and diagnosis.

## MATERIALS AND METHODS

### Tissue specimens

Thirty-six postmenopausal patients with osteoporosis (Osteoporosis group) and 36 postmenopausal patients without osteoporosis (Control group) were identified at the Hospital attached to University between May 2016 and May 2019 and enrolled in this study. Peripheral blood was collected and total RNA was isolated. The Ethics Committee of University approved this study, which was carreid out in compliance with the Helsinki Declaration. All osteoporosis and control patients provided informed consent prior to the start of the study.

### Cell culture

MC3T3-E1 cells were purchased from the American Type Culture Collection (Manassas, VA, USA). The cells were grown in α-modified minimal essential medium (a-MEM; Invitrogen, Carlsbad, CA, USA) including 10 % fetal bovine serum (FBS; Gibco, Grand Island, NY, USA), 50 mg/ml streptomycin, and 50 IU/ ml penicillin in a humidified cell incubator containing 5% CO_2_ at 37° C. Thereafter, 50 mg/L ascorbic acid and 10 mM β-glycerophosphate were added to the medium to obtain an *in vitro* model of osteogenic differentiation.

### Cell transfection

MC3T3-E1 cells were transiently transfected with miR-483-5p mimic and inhibitor to increase or decrease the miR-483-5p expression. The inhibitor and mimic were acquired from GenePharma Co., Ltd. (Shanghai, China) and were transfected utilizing Lipofectamine 2000 (Invitrogen, Carlsbad, CA). The PI3K/AKT signaling pathway was inhibited by treating MC3T3-E1 cells with 10 μM LY294002 (Merck Millipore, Germany) before the miR-483-5p mimic transfection. To overexpress SATB2 in MC3T3-E1 cells, the lentiviral vector system was used. The complete SATB2 coding sequences were amplified and inserted into the core plasmid pLVX-Puro. For the lentivirus production, Lipofectamine 2000 was employed to transfect the recombinant plasmid into HEK293T cells following the manufacturer’s guidelines. The virus supernatant was collected 48 h later and concentrated by ultracentrifugation for 10 min (4,000 g at 4° C). For cell infection, cells were planted in 6-well plates (5×104 cells/well). To explore the correlation between SATB2 and miR-483-5p, MC3T3-E1 cells were co-transfected with miR-483-5p mimic and SATB2-overexpressed lentiviruses (Lv-SATB2). The transfection efficiency was detected 48 h after transduction.

### Cell viability and LDH detection

Cell viability was analyzed employing CCK-8 assay. Briefly, CCK-8 reagent (10 ml) was added to the MC3T3-E1 cells (5×103 cells/well) for 2 h, and then the absorbance at 450 nm wavelength was determined in a microplate reader. For LDH detection, the supernatant of MC3T3-E1 cells were collected and pyruvic acid, coenzyme I and 2,4-dinitrophenylhydrazine were added sequentially to the cells. The absorbance at 450 nm wavelength was also determined in a microplate reader.

### Luciferase reporter assay

MiR-483-5p targets were predicted using the TargetScan 7.1 software (http://www.targetscan.org/). The mutant (MUT) or wild-type (WT) 3’-UTR sequence of SATB2 was cloned into the dual luciferase reporter vectors (Promega, Madison, WI, USA). Then, WT or MUT SATB2 3’UTR plasmid along with miR-483-5p mimic/inhibitor or negative control (NC) was co-transfected into MC3T3-E1 cells. The transfected cells were collected 48 h after transfection and the luciferase activities were determined through a dual-luciferase reporter assay kit (Promega, Madison, WI, USA). Each experiment was carried out in triplicate.

### Ovariectomized osteoporosis model

Animals experiments were performed in accordance with protocols accepted by the Ethical Review Committee of University and following the Guide for the Care and Use of Laboratory Animals (National Institutes of Health, 8th edition). Six-week old female Sprague-Dawley rats (weight, 180 ± 20 g) were acquired from Beijing HFK Bioscience Co., Ltd. and maintained in polycarbonate cages under controlled lighting (12-hour light/dark cycle), humidity (55 ± 5%) and temperature (22 ± 2° C). After a week of adaptation, the rats were randomly divided using a table of random numbers into four groups (n=12): control (sham), model, miR-483-5p inhibitor and si-SATB2. The rats in the model group were subjected to unilateral ovariectomy. Briefly, the rats were anesthetized with 2% of isoflurane and a unique longitudinal skin incision was performed on the dorsal midline around the position of kidneys. Then, the ovary was ligated and excised. The rats in the sham group were subjected to incision and ovary exposure, but not to excision of the ovary. After 4 weeks of operation, 100 μl of miR-483-5p inhibitor and/or small interfering RNA of SATB2 (si-SATB2) were injected subcutaneously for another four weeks (one injection per week). The rats were anesthetized and the femurs were removed after the last injection.

### Bone mineral density and biomechanical parameters analysis

The left femurs were excised from the rats and Lunar DPX-IQ Dual Energy X-ray absorptiometry (DEXA) with a PIXImus II densitometer (Lunar Corporation, Madison, WI) was used to measure the BMD levels. A computer-controlled mechanical testing machine (SANS-10404043, Shenzhen, China) was employed to detect the elasticity modulus, maximum load and maximum bending stress in accordance with the three-point bend testing. A sample space of 23 mm and plunger speed of 2.0 mm/min were the texting conditions.

### Real-time quantitative reverse transcription PCR (qRT-PCR)

Total RNA from cells and tissues was isolated employing the TRIzol reagent and transformed to cDNA using the Bestar™ qPCR RT kit (DBI Bioscience, Ludwigshafen, Germany) in accordance with the manufacturer's instructions. SYBR-Green PCR Master Mix (Bio-Rad, Hercules, CA, USA) was performed on the 7500 Real-time PCR System (Applied Biosystems) to evaluate the relative SATB2 and miR-483-5p levels. The 2^-ΔΔCt^ method was employed to measure the mRNA expression, which was normalized to GAPDH or U6. All the experiments were carried out in triplicate. The primer sequences were: miR-483-5p, forward, 5'-TCAACGGGACAGACAAAGAT-3' and reverse, 5'-CTCAGGATGGAGCAGAGGG-3'; U6, forward, 5'-TATGGCTCCTTTCACCTG-3' and reverse, 5'-CCTGGCAGACAGTCAGAA-3'; SATB2, forward, 5’-GAGGAAGGCTTGGGAGTA-3’ and reverse, 5’-GGGCAGCAGAGCTATGTG-3; GAPDH, forward, 5’-GTCAACGGATTTGGTCGTAT-3’ and reverse, 5’-TTCTCCATGGTGGTGAAGAC-3’.

### Western blot assay

Total proteins were extracted by RIPA lysis buffer (Beyotime, Jiangsu, China). The protein concentration was quantified through a BCA Kit (Beyotime). Each 20 μg of protein was separated by 10% SDS-PAGE and then transferred to PVDF membranes (Millipore, Billerica, MA, USA). The membranes were blocked using non-fat milk (5%) at room temperature for 1 h, which were subsequently incubated at 4° C overnight with the following primary antibodies: anti-OPG (1:100; Cell Signaling Technology, USA), anti-RANKL (1:1000; Abcam, USA), anti-RUNX2 (1:1000; CST), anti-BMP2 (1:1000; CST), anti-p-PI3K (1:1000; Abcam), anti-t-PI3K (1:1000; Abcam), anti-p-AKT3 (1:1000; Abcam), anti-t-AKT3 (1:1000; Abcam), anti- p-GSK3β (1:1000; Abcam) anti-β-catenin (1:5000; Abcam). Then, the secondary antibody was added and incubated with the membranes for 2 h. The signal of the protein bands was visualized using ECL Plus reagents and their densities were evaluated by the Quantity One software (Bio-Rad Laboratories). All the assays were performed in triplicate.

### Statistical analysis

Statistical analyses were carried out with SPSS software (v. 22.0; SPSS Inc., Chicago, IL). The data were showed as mean ± standard deviation (SD). The differences between multiple comparisons were evaluated by one-way analysis of variance (ANOVA) followed by a post hoc Tukey test. The association between SATB2 and miR-483-5p was analyzed by Pearson analysis. P < 0.05 indicated statistically significant.
